# Systemic lupus erythematosus complicated by Crohn’s disease with rectovaginal fistula

**DOI:** 10.1186/s12876-021-01801-w

**Published:** 2021-05-08

**Authors:** Heng Yeh, Ren-Chin Wu, Wen-Sy Tsai, Chia-Jung Kuo, Ming-Yao Su, Cheng-Tang Chiu, Puo-Hsien Le

**Affiliations:** 1 School of Medicine, College of Medicine, Chang Gung University, Taoyuan, Taiwan; 2 Department of Pathology, Chang Gung Memorial Hospital, Linkou Branch, Taoyuan, Taiwan; 3 Department of Colon and Rectal Surgery, Linkou Chang Gung Memorial Hospital, Taoyuan, Taiwan; 4 Department of Gastroenterology and Hepatology, Chang Gung Memorial Hospital, Linkou Branch, Taoyuan, Taiwan; 5 Department of Gastroenterology and Hepatology, New Taipei City Municipal Tucheng Hospital (Chang Gung Memorial Hospital, Tucheng Branch), Tucheng, Taiwan; 6 Liver Research Center, Chang Gung Memorial Hospital, Linkou Branch, Taoyuan, Taiwan; 7 Taiwan Association of the Study of Small Intestinal Disease, Taoyuan, Taiwan

**Keywords:** Rectovaginal fistula, Crohn’s disease, Cryptococcal pneumonia, Systemic lupus erythematosus, Vedolizumab, Case report

## Abstract

**Background:**

Systemic lupus erythematosus (SLE) is a multisystemic autoimmune disease, and few cases combine with Crohn’s disease. We present the first SLE patient concurrent with Crohn’s disease and rectovaginal fistula. She was successfully treated with vedolizumab and surgical intervention. Besides, she also had a rare opportunistic infection, cryptococcal pneumonia, in previous adalimumab treatment course.

**Case:**

A 57 year-old female had SLE in disease remission for 27 years. She suffered from progressive rectal ulcers with anal pain and bloody stool, and Crohn’s disease was diagnosed. She received adalimumab, but the lesion still progressed to a rectovaginal fistula. Besides, she suffered from an episode of cryptococcal pneumonia under adalimumab treatment course. Therefore, we changed the biologics to vedolizumab, and arrange a transverse colostomy for stool diversion. She had clinical remission without active inflammation, but the fistula still persisted. Then, she received a restorative proctectomy with colo-anal anastomosis and vaginal repair. Follow-up endoscopy showed no more rectal ulcers or fistula tracts, and contrast enema also noted no residual rectovaginal fistula.

**Conclusion:**

When a SLE patient had unusual rectal ulcers, Crohn’s disease should be considered. Biologics combined with surgical intervention is an optimal solution for Crohn’s disease with rectovaginal fistula. Although cryptococcal pneumonia is a rare opportunistic infection in the biological treatment, we should always keep it in mind.

## Background

Systemic lupus erythematosus (SLE) is an autoimmune disease affecting mostly young female adults and characterized by skin lesions, arthritis, hematologic disorders, multi-organ involvement and presence of autoantibodies [1]. The 53% patients with SLE had lupus enteritis, and 8–40% have digestive system involvement [2]. However, the concurrence of SLE and Crohn disease (CD) is uncommon [3, 4].

Rectovaginal fistula (RVF) is a rare, but troublesome complication of Crohn’s disease (CD). Although some patients attained complete closure with 6 mercaptopurine (6-MP), tacrolimus or infliximab, more than 60% patients still required surgical repair [5–8]. Vedolizumab (VDZ) is a humanized monoclonal antibody (IgG1) to α4β7 integrin with demonstrated efficacy in the treatment of patients with moderate to severe inflammatory bowel disease. The exploratory analyses of data from GEMINI 2 showed beneficial effect of VDZ treatment for fistulizing Crohn’s disease (CD), but there was no statistical significance [9]. The subgroup of patients with RVF was not analyzed separately, and no other studies reported the efficacy of vedolizumab in rectovaginal fistula closure.

Although there are several opportunistic infections reported in inflammatory bowel disease (IBD), only one patient treated with prednisone, azathioprine and adalimumab had cryptococcal pneumonia [10]. We report a SLE patient who developed CD with RVF. Besides, she experienced an episode of pulmonary cryptococcosis in previous adalimumab treatment course.

## Case presentation

**Fig. 1:**
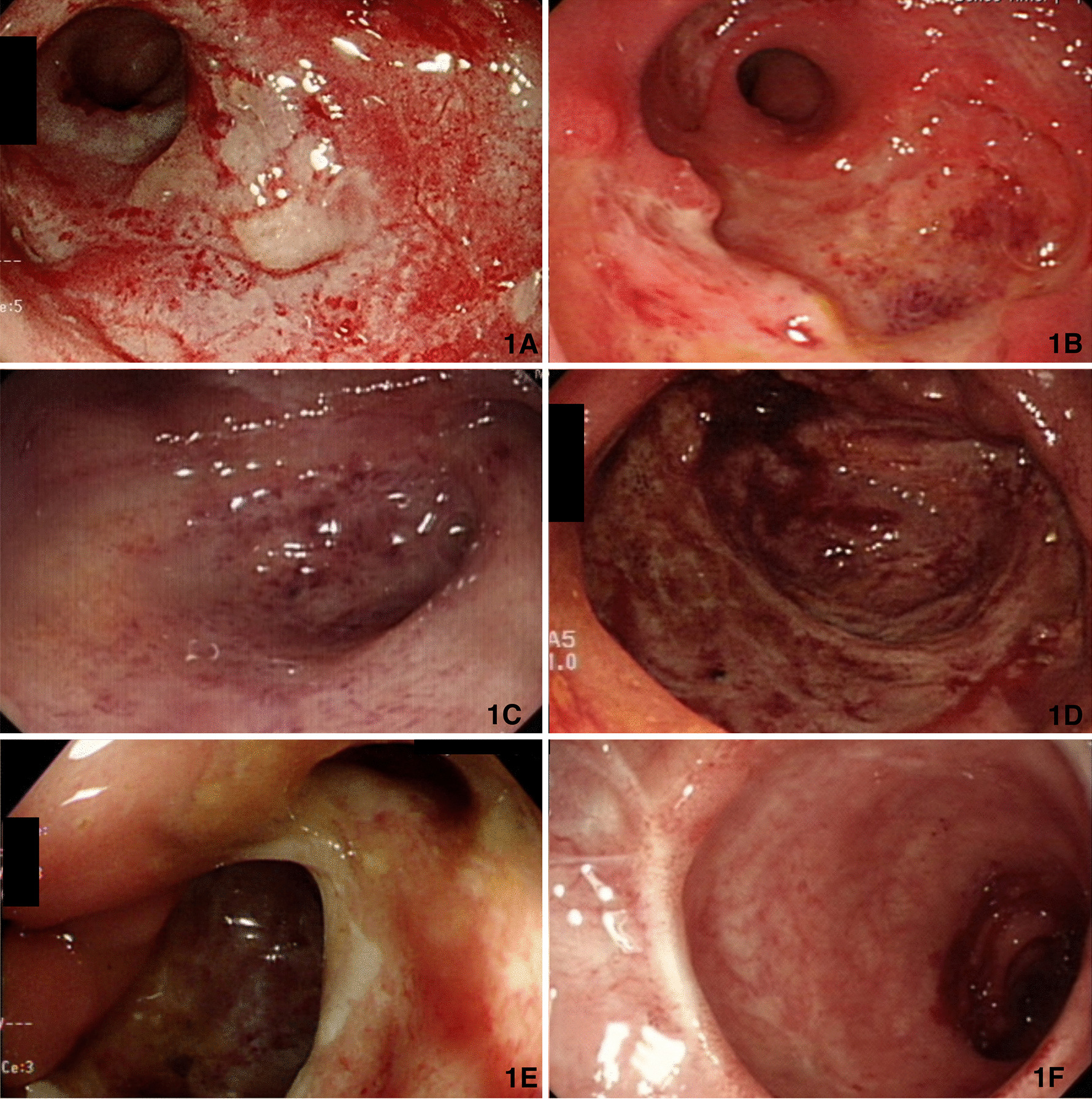
Endoscopic presentations. **a** Initial endoscopic presentation: several shallow rectal ulcers, **b** 2 months after hydrocortisone and mesalazine enema: huge and deep rectal ulcers, **c** 1.5 months after anti-viral therapy: healing ulcers, **d** progressive huge rectal ulcer 3 months later, **e** rectovaginal fistula **f** No residual fistula tract or inflammation after vedolizumab and surgical intervention

This 57-year-old female had SLE under methotrexate 7.5 mg/week and prednisolone 7.5 mg/day treatment with C3 level 65.00 mg/dL (Normal range 90–180 mg/dL) and C4 level 18.20 mg/dL (Normal range 10–40 mg/dL) for 27 years. She received a right total hip replacement due to right hip avascular necrosis on September, 2006. She complained progressive anal pain with bloody stool for months. There was no fever, no chills, no abdominal pain, no oral or genital ulcers and negative pathergy test. Laboratory exam showed positive anti-SSA antibody (Ab) and cytomegalovirus (CMV) IgG, but negative anti-SSB Ab, anti-RNP Ab, Amebic AB, CMV IgM, CMV DNA, Epstein–Barr virus (EBV) DNA, blood culture, stool cultures (Salmonella, Shigella, Campylobacter, Clostridium difficile), Clostridium difficile toxin or Rotavirus. Sigmoidoscopy revealed rectal ulcers, 0.5–0.8 cm, 3–10 cm level above anal verge (Fig. 1a), and histology revealed the features of acute inflammation (lymphoplasmacytic infiltration) and chronic inflammation (crypt branching, dropout and shortfall), which were compatible with inflammation bowel disease without evidence of CMV infection or vasculitis. She received hydrocortisone and mesalazine enema once daily. Bloody stool subsided and anal pain improved initially, but the condition progressed after 2 weeks of treatment.

**Fig. 2:**
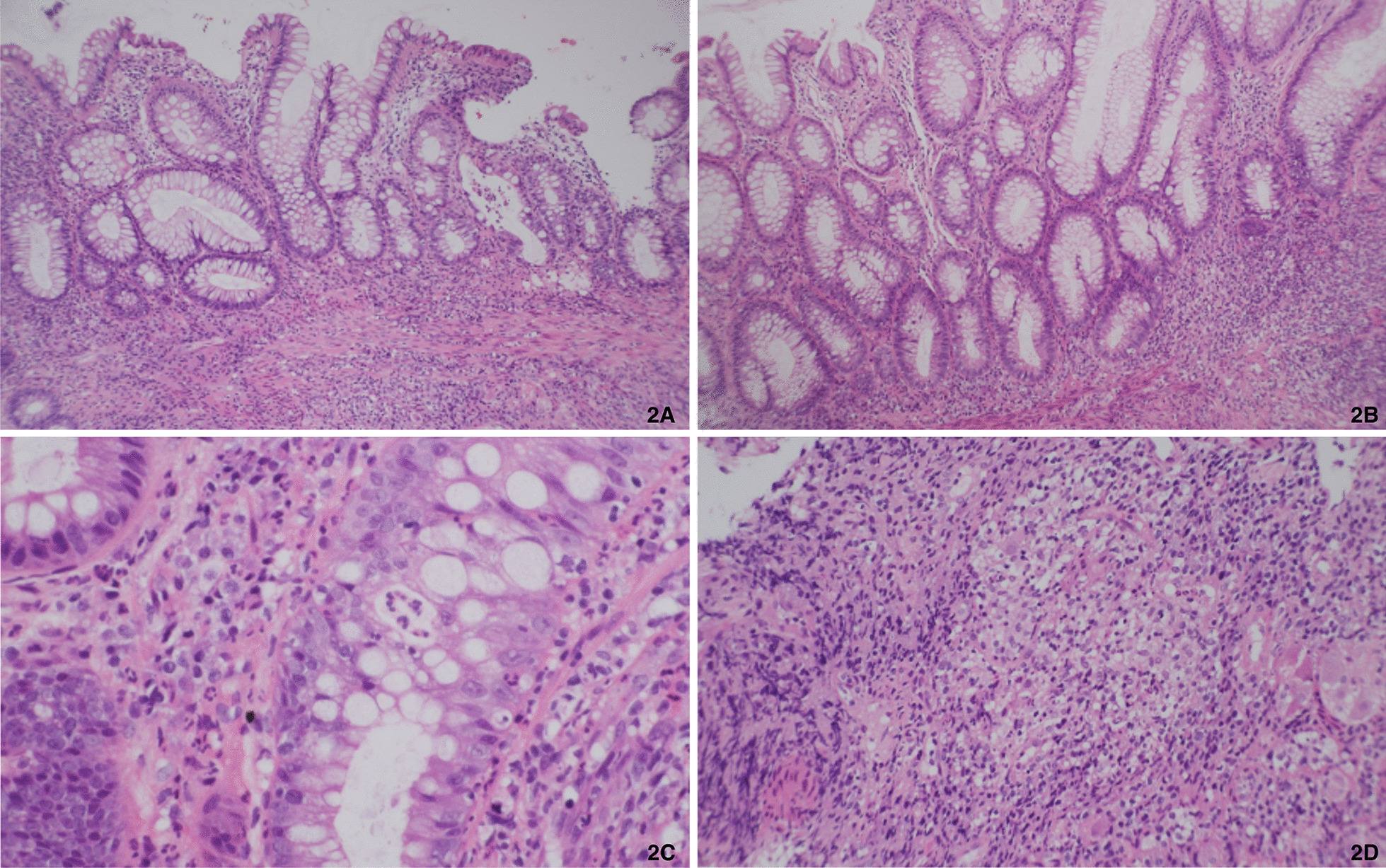
Pathology findings. **a** acute inflammation with lymphoplasmacytic infiltration, **b** Features of chronicity: crypt branching, crypt dropout, crypt shortfall, **c** crypt abscess, **d** non-caseating granulomas

Colonoscopy showed huge and deep rectal ulcers 2 months after first endoscopic examination, and histology revealed acute on chronic inflammation with crypt abscess, granuloma and positive immunohistochemical staining for CMV (Fig. 2). Ganciclovir (200 mg IV Q12h) was prescribed for 2 weeks followed by valganciclovir (450 mg/tab 1PC BID) for another 6 weeks to treat CMV colitis and hydroxychloroquine (200 mg/tab 1PC BID) and prednisolone (10 mg daily) were given for SLE control. The follow-up sigmoidoscopy 6 weeks after anti-viral treatment showed healing ulcers (Fig. 1c) and repeated immunohistochemical staining for CMV were both negative. We kept valganciclovir, hydroxychloroquine and prednisolone using. However, she had recurrent pain with bloody stool, and sigmoidoscopy showed a huge progressive rectal ulcer 3 months later (Fig. 1d). Blood CMV Ag, CMV IgM, EBV Ag, Clostridial difficile toxin and rectal tissue IHC staining for CMV were all negative. Adalimumab was prescribed for Crohn’s disease.

**Fig. 3:**
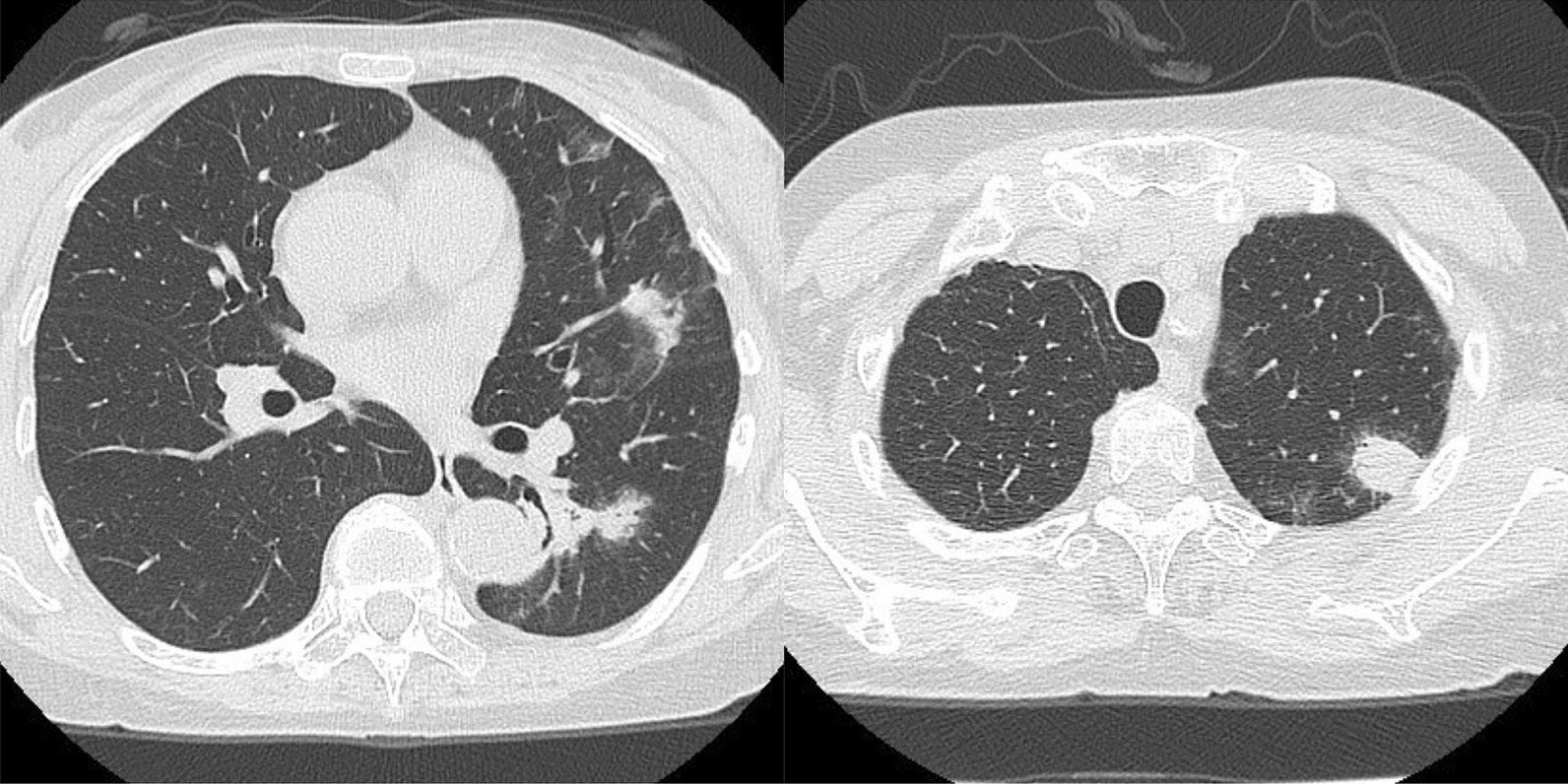
Computer tomography of chest showing poor-defined margin nodules in left upper lobe and left lower lobe, and pathology revealed Cryptococcus infection

**Fig. 4:**
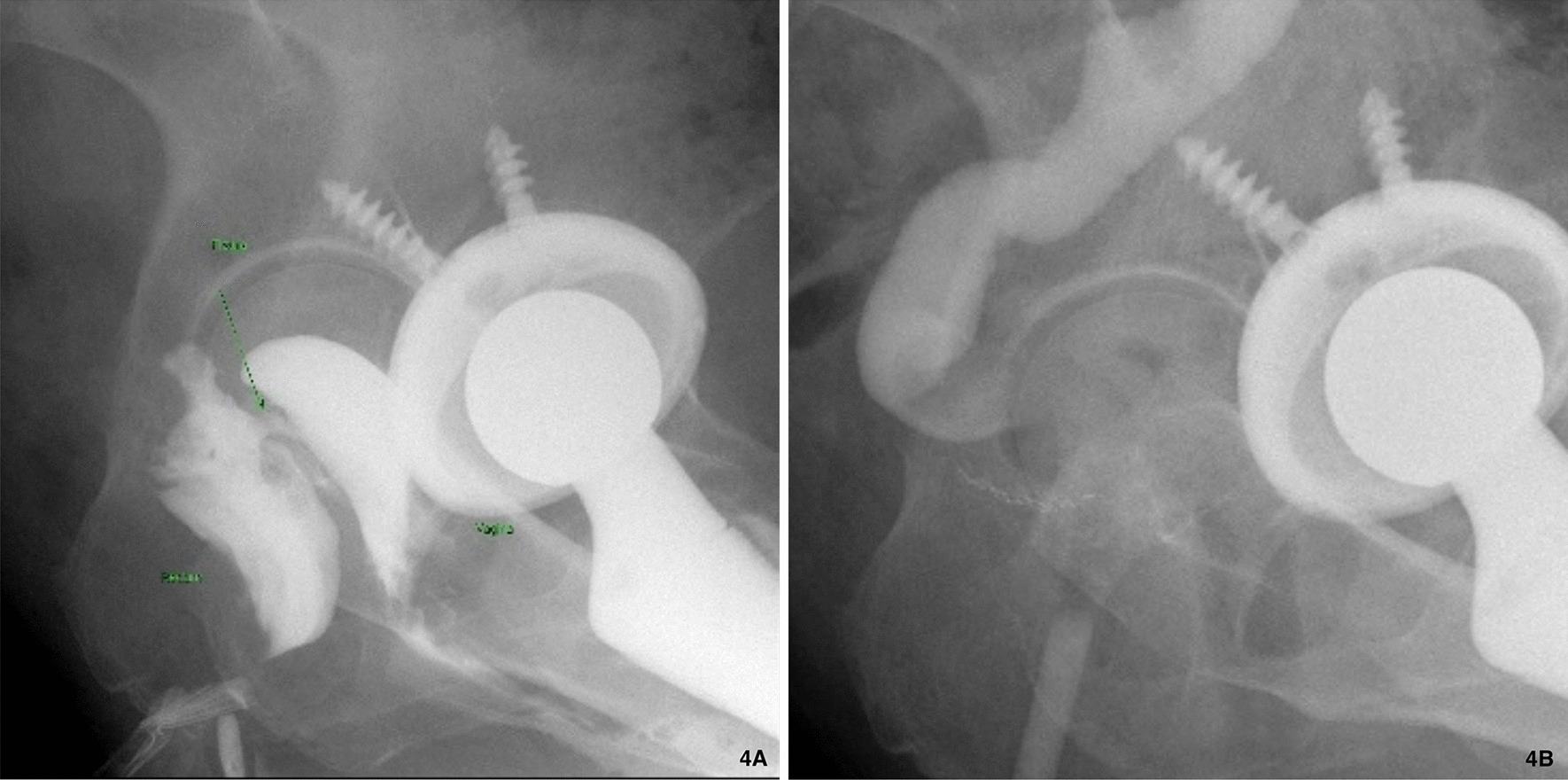
Fistulogram and Contrast enema: Injection of water soluble contrast medium into rectum via 16 Fr. Foley **a** Before vedolizumab treatment: presence of rectovaginal fistula (6 mm in length and 3 mm in diameter) over anterior wall of rectum about 3.5 cm above anus. **b** One year after vedolizumab treatment and transverse colostomy: no residual rectovaginal fistula was noted

Besides, she had an episode of cryptococcal pneumonia (Fig. 3) proved by lung biopsy and cryptococcal Ag 1:64 3 months after receiving adalimumab therapy. She mentioned the exposure history to pigeon manure because her neighbor was a pigeon fancier. Amphotericin B and flucytosine were prescribed, and symptoms improved. She also complained progressive bloody stool and noted some stool passage from vagina under adalimumab treatment course. Sigmoidoscopy (Fig. 1e), fistulogram (Fig. 4a) and MRI all confirmed the rectovaginal fistula. Then, we arranged a transverse colostomy and shift the biologics to vedolizumab. She had clinical remission 2 months later, and we kept vedolizumab using. One year later, Sigmoidoscopy revealed no inflammation, and she received a restorative proctectomy with colo-anal anastomosis and vaginal repair. Follow-up endoscopy showed no more rectal ulcers or fistula tracts (Fig. 1f), and contrast enema (Fig. 4b) also noted no residual rectovaginal fistula 10 months later.

## Discussion and conclusion

SLE is a multisystemic autoimmune disease with variable presentations. Gastrointestinal (GI) manifestations are noted in 50% SLE patients, including lupus enteritis, pancreatitis and peritonitis [11, 12]. However, only few SLE patients complicated with rectal ulcers were reported, and some of them were successfully treated with intravenous steroid, tacrolimus, cyclophosphamide and surgery [13–18]. Although Crohn’s disease mainly involves gastrointestinal tract, 6% patients have extraintestinal manifestations (EIM) at diagnosis, and 25–47% patients experience EIM in lifetime [19–21]. The most common EIM include arthritis, skin involvement, and ocular manifestations, etc. Besides, it sometimes combined with other autoimmune diseases. It’s not easy to differentiate between SLE with GI involvement and Crohn’s disease. There are still some hints to make the differential diagnosis according to previous study results [3, 4]. Compared to SLE, CD presents more frequently as diarrhea, hematochezia and perianal lesions. In the perspective of image studies, lupus enteritis is characterized with segmental bowel dilatation and air fluid level, fingerprint sign and false obstruction. CD has comb sign and skipped lesions over the whole alimentary tract. When a SLE patient has GI symptoms, but the clinical presentations and image features are more suggestive of CD, we should arrange endoscopic exam with biopsy to confirm the diagnosis. If the colonoscopy shows skipped, longitudinal ulcers with cobble stone like mucosal changes, CD is the preferred diagnosis. Furthermore, non-caseating granuloma and crypt abscess are more specific for CD, and vasculitis or vascular ischemic changes are more common in SLE. In this patient, she had hematochezia, rectal ulcers, RVF, but without extra-intestinal SLE signs. Histology revealed acute on chronic inflammation with non-caseating granuloma and crypt abscess without evidences of vasculitis or ischemic changes. She had a poor response to intensive SLE treatment even with normal C3, C4 levels. Therefore, SLE concurrent with CD and RVF was diagnosed.

TNF-α inhibitors are effective in CD treatment,but it also increases the risk of opportunistic infections, including Pneumocystis pneumonia, cryptococcal septicemia and meningitis, Klebsiella septicemia, and etc. [22, 23]. In this case, she had a tissue proved Cryptococcal pneumonia with nodules formation 3 months after the initiation of adalimumab. To the best of our knowledge, there was only one similar case reported before [10]. Blood cryptococcal antibody test, pulmonary computer tomography and biopsy were crucial to make early diagnosis of this severe fatal opportunistic infection under TNF-α inhibitors treatment.

Rectovaginal fistula occurs in 3–5% of all female patients diagnosed with CD [6]. There was a cohort study collecting total 47 Crohn’s disease patients complicated with genital fistula and total 35 patients in the study are complicated with RVF. The major symptoms of the genital fistula included feces and gas passage through the genital tract, and some patients suffered from infection and dyspareunia. The mean time from the diagnosis of Crohn’s disease to the appearance of the genital fistula was 102 + − 96.8 months in the study [24]. Although the recent systematic review showed increasing role in biological treatments of fistula, the combination of biologics and surgical intervention is most effective way in this condition [6]. In this case, surgical repair could lead to anastomotic leakage and intraabdominal abscess formation due to severe inflammation. After good inflammation control with vedolizumab, operation could be performed safely and smoothly.

This SLE patient concurrent with CD and rectovaginal fistula who was successfully treated with vedolizumab and surgical intervention, but she experienced an episode of cryptococcal pneumonia during the previous adalimumab treatment. When a SLE patient had rectal ulcers, Crohn’s disease should be considered after ruling out infectious disease. Biologics combined with surgical intervention is an optimal solution for Crohn’s disease with rectovaginal fistula. Cryptococcal pneumonia is a rare but fatal opportunistic infection in patients receiving biological treatment, we should always keep the disease in mind.

## Data Availability

Not applicable.

## Abbreviations

SLE: Systemic lupus erythematosus
CD: Crohn’s disease
RVF: Rectovaginal fistula
6-MP: 6-Mercaptopurine
VDZ: Vedolizumab
IBD: Inflammatory bowel disease
Ab: Antibody
Ag: Antigen
CMV: Cytomegalovirus
EBV: Epstein–Barr virus
GI: Gastrointestinal
EIM: Extra-intestinal manifestations
TNF: Tumor necrosis factor
